# J-Shaped Association of Tomato Intake with New-Onset Hypertension in General Adults: A Nationwide Prospective Cohort Study

**DOI:** 10.3390/nu14224813

**Published:** 2022-11-14

**Authors:** Dan Zhao, Zezhong Tian, Ying Liang, Hong Chen, Zhiying Fan, Zhihao Liu, Suming Dai, Meitong Liu, Huiying Kuang, Yan Yang

**Affiliations:** 1 School of Public Health (Shenzhen), Shenzhen Campus of Sun Yat-sen University, Sun Yat-sen University, Shenzhen 518107, China; 2 Guangdong Provincial Key Laboratory of Food, Nutrition, and Health, Sun Yat-sen University, Guangzhou 510080, China; 3 Guangdong Provincial Engineering Technology Center of Nutrition Transformation, Sun Yat-sen University, Guangzhou 510080, China; 4 Shangyu Center for Disease Prevention and Control, Shaoxing 312300, China; 5 School of Public Health, Baotou Medical College, Baotou 014000, China

**Keywords:** new-onset hypertension, dietary tomato, primary prevention, dose-response relationship

## Abstract

We aim to examine the prospective association between the intake of dietary tomatoes and the risk of new-onset hypertension and its modifiable factors in general adults. A total of 11,460 adults without hypertension from the China Health and Nutrition Survey (CHNS) were enrolled, with follow-up beginning in 1997 and ending in 2015. Dietary tomato intake was measured by three consecutive 24-h dietary recalls combined with a household food inventory. The study outcome was new-onset hypertension, defined as systolic blood pressure ≥ 140 mmHg or diastolic blood pressure ≥ 90 mmHg or diagnosed by physicians or under anti-hypertensive treatment during the follow-up. Finally, 4015 subjects developed new-onset hypertension during 92,335.5 person-years of follow-up. After multivariate adjustment for dietary and non-dietary risk factors, hazard ratios for increased consumption of dietary tomatoes were 0.42 (95% confidence interval, 0.37–0.47), 0.51 (0.46–0.57), and 0.82 (0.74–0.92) compared with non-consumers. Overall, cubic spline regression suggested a novel J-shaped association between dietary tomato intake and new-onset hypertension, with the lowest risk observed at approximately 10 to 13 g/day (*p* < 0.001 for curvature). Moreover, the association between dietary tomato intake and risk of new-onset hypertension was stronger in females or individuals who refrained from smoking or drinking (*p* = 0.024, *p* = 0.043, and *p* = 0.044 for interaction, respectively).

## 1. Introduction

Hypertension is one of the main modifiable risk factors for cardiovascular disease (CVD), which is the leading cause of death (8.5 million deaths each year) and disease burden worldwide [1,2]. There is a rapid increase in the prevalence of hypertension in developing countries. Nationally representative survey data show a prevalence of hypertension of 23.2% in Chinese adults [3], and approximately 244.5 million Chinese adults are currently hypertensive. Therefore, developing effective strategies to inform the primary prevention of hypertension will help to achieve large health gains in the general population. Recently, the effects of lifestyle modifications, in particular dietary management, have tended to be one of the common and cost-effective approaches for the prevention of hypertension [4,5].

The epidemiological evidence suggests that the high consumption of fruits and vegetables may reduce hypertension risk [6]. Tomatoes, one of the most frequently consumed fruit/vegetables, have been widely recognized as healthy food. Multiple randomized controlled trials (RCT) have shown that supplementation of tomato constituents, such as lycopene [7], specific flavonoid compounds [8,9], and ascorbic acid [10], have beneficial effects on blood pressure. However, very few studies have directly addressed the effects of tomatoes, as one kind of daily food, on human health. A previous short-term RCT with 32 type 2 diabetic patients found that dietary raw tomatoes have the potential to reduce blood pressure [11]. Although RCTs can generate powerful evidence for the effects of interventions, short-duration interventions and insufficient sample sizes are limited to intermediate risk factors as endpoints and are unable to explore the potential modifying factors. Additionally, their results conducted under ideal and controlled circumstances are difficult to generalize to larger, more inclusive populations [12]. Hitherto, the limited number of prospective cohort studies have found inverse associations between dietary tomato intake and the risk of developing several major chronic diseases, including CVD [13], hepatocellular carcinoma [14], bladder cancer [15], and mortality [16]. However, it is unclear whether dietary tomato intake is associated with new-onset hypertension in general adults. Furthermore, to date, the optimal intake of dietary tomatoes for preventing new-onset hypertension in the general population has not been explored yet.

To address these research gaps, we used large-scale cohort data to prospectively examine the association of dietary tomato intake with new-onset hypertension, clarify a dose-response relationship in the primary prevention of hypertension among the general population and explore modifying factors that might affect these associations.

## 2. Materials and Methods

### 2.1. Study Design and Population

The present study is based on the China Health and Nutrition Survey (CHNS), which is an ongoing, prospective cohort study established in 1989. Nowadays, 10 surveys undertaken between 1989 and 2015 have already been completed. The survey protocols, instruments, and the process for obtaining informed consent were approved by institutional review boards at the University of North Carolina, Chapel Hill (Chapel Hill, NC, USA), and the National Institute of Nutrition and Food Safety, China Center for Disease Control and Prevention (Beijing, China), and each participant provided written informed consent. This cohort has been described in detail elsewhere [17].

In the current study, we used data on 7 waves of CHNS from 1997 to 2015; the general population, who are less likely to receive invasive or costly examinations in clinics, was included. Accordingly, we excluded participants who were less than 18 years old, who were pregnant, or who had a diagnosis of a history of stroke, myocardial infarction, or any type of tumor at baseline because the diagnoses of these conditions can lead to changes in diet and lifestyle. Furthermore, participants with implausible dietary energy data (men, >8000 or <800 kcal/day; women, >6000 or <600 kcal/day) and those with only one study round were also excluded. After exclusions, our analysis included a total of 11,460 participants (Supplementary Figure S1).

### 2.2. Dietary Nutrient Intakes

Dietary data were assessed for three consecutive days by trained nutritionists using dietary recalls in combination with a weighing and measuring technique in each survey round. Briefly, individual diet intake was assessed through 24-h recalls both away from home and at home for three consecutive days. The validity of the 24-h dietary recall in measuring food intake has been described in detail previously [18]. Household food consumption was determined by a weighing and measuring technique over the same three days. Three-day average intakes of dietary tomatoes, vegetables and fruits in each round were calculated. The cumulative average consumption of each food from baseline to the last visit before the date of new-onset hypertension or the end of follow-up was adopted to better represent the long-term diet and minimize within-individual variation. Dietary tomato intake was calculated by summing the intakes of raw tomatoes, canned tomatoes, and ketchup.

### 2.3. Assessments of Covariates

We used a validated questionnaire to assess demographic and lifestyle information, including age, sex, smoking and drinking status, education level, and region. For smoking, we combined data on current and past smoking. Body height and weight were measured following a standard procedure with calibrated instruments. Body mass index (BMI) was calculated as weight in kilograms divided by height in meters squared. Systolic blood pressure (SBP) and diastolic blood pressure (DBP) were measured using a standard mercury sphygmomanometer by well-trained physicians. Measures were used on the right arm after 10 min of seated rest and were repeated three times at an interval of 3–5 min. The mean of the three measurements was used in the analysis. Physical activity was assessed based on the time and intensity of occupational, household, transportation and leisure activities by a validated self-reported questionnaire. Intakes of total carbohydrates, total proteins and total fats were calculated on the basis of the Chinese food composition table (FCT).

### 2.4. Assessment of Outcomes

New-onset hypertension was the outcome; it was identified through a diagnosis by physicians, the current use of anti-hypertensive drugs, or by mean SBP ≥ 140 mmHg or diastolic BP ≥ 90 mmHg.

The study baseline was the year of each participant’s first entry into the survey with a complete dietary record. Person-years of follow-up for each participant were calculated from baseline until the first diagnosis with hypertension (the middle date between the survey of the first diagnosis and the nearest survey before), the last survey round before the participant was lost to follow-up, or until being censored at the end of the follow-up period, whichever came first.

### 2.5. Statistical Analysis

Baseline characteristics are presented as mean (SD) or interquartile ranges (IQRs) for continuous variables, and categorical variables were presented as numbers (percentages). Dietary tomato intake was categorized into four groups, group 1: no tomato intake; group 2: 0 to ≤13.3 g/day; group 3: 13.3 to ≤33.3 g/day; group 4: >33.3 g/day. We used the χ^2^ test or ANOVA to examine participant characteristics according to dietary tomato intake.

A multivariable Cox proportional hazards model was used to estimate hazard ratios (HR) and 95% confidence intervals for the association between dietary tomato intake and new-onset hypertension. We assessed the proportional hazards assumption and identified no violation. The lowest dietary tomato intake group (group 1) was used as the reference group for all analyses. In the final Cox models, we adjusted for the following potential confounders: age at baseline (continuous), sex (male/female), BMI (continuous), smoking (yes/no) and drinking status (yes/no), education (middle school or below, high school or college or above), residence (urban or rural), marital status, physical activity (continuous), as well as cumulative average vegetables (continuous), fruits (continuous), and cumulative average energy intake (continuous). Additionally, dose-response relationships were examined with restricted cubic spline (RCS) Cox regression between tomato intake and new-onset hypertension. Based on the corrected Akaike information criterion, we selected the knots as 4. The likelihood ratio test of the cross-product terms between tomato intake and effect modifiers was used to test the significance of interactions. Tests for linear trends were performed considering the category of intake as a continuous variable in the model.

Stratified analyses were performed to evaluate the possible effect modification according to sex (male or female), age (<45 or ≥45 years), SBP (<120 or ≥120 mmHg), BMI (<24 or ≥24 kg/m^2^), smoking status (no or yes), drinking status (no or yes), fat intake (<62.9 or ≥62.9 g/day (median)), protein intake (<65.2 or ≥65.2 g/day (median)), and carbohydrate intake (<312.8 or ≥312.8 g/day (median)).

Furthermore, a series of sensitivity analyses were used to test the robustness of the findings. First, in a lag analysis, we excluded participants who developed new-onset hypertension events during the first two years of follow-up to minimize the influence of reverse causation. Secondly, raw tomato consumption was examined with respect to the risk of new-onset hypertension. Third, the follow-up person-time was calculated from baseline until the first hypertension diagnosis. Fourth, considering the missing values during follow-up, multiple imputation models by chained equations with five data sets were used to obtain robust HRs with 95% CIs [19]. Detailed information on the number of missing covariates is shown in Table S1. Fifth, we examined whether the results would be changed if tomato consumption was divided into five groups (group 1: no tomato intake, group 2: 0–11.1 g/day, group 3: 11.1–20 g/day, group 4: 20–35 g/day, group 5: >35 g/day). Lastly, the E-value [20] was used to test the magnitude of an unmeasured confounding factor that could affect the association by random chance.

A 2-tailed *p* < 0.05 was considered to be statistically significant in all analyses. Analyses were performed using R software version 4.1.3 (http://www.R-project.org/, accessed on 10 April 2022).

## 3. Results

### 3.1. Baseline Characteristics

As illustrated in the flowchart, 27,887 participants completed a dietary record at baseline (Figure S1). Of these, a total of 11,460 participants were included in the final study. Among these included participants, 7075 (61.7%) did not consume tomatoes. The average age of the study population was 41.7 (SD, 13.9) years. People with a higher intake of tomatoes were more likely to be older, male and married and tended to have a higher BMI, higher systolic and diastolic blood pressure, higher education degree, higher baseline alcohol consumption rate, higher total carbohydrate, fat, protein intake, and were characterized by a higher intake of fruits and vegetables and less physical activity (all *p* for difference < 0.05). Detailed characteristics of participants according to the amount of tomato intake and sex are provided in Table 1 and Supplementary Table S2, respectively.

### 3.2. Association between Dietary Tomato Intake and New-Onset Hypertension

**Figure 1:**
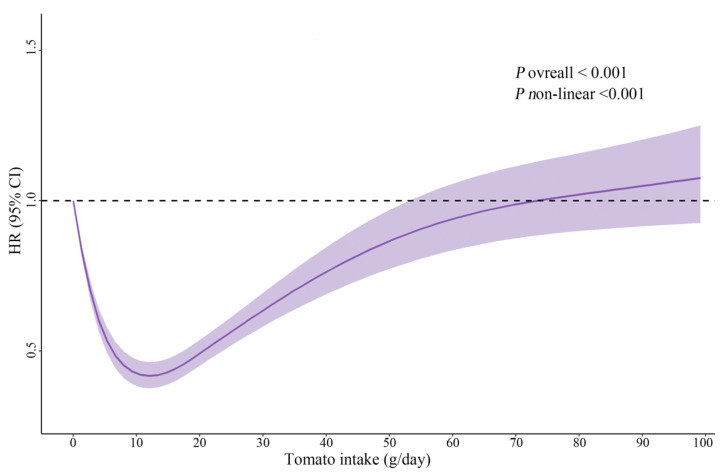
Dose-response relationship between dietary tomato intake and new-onset hypertension. Restricted cubic spline Cox proportional hazard regression model with 4 knots was adjusted for age at baseline (continuous), sex (male/female), BMI (continuous), smoking (yes/no) and drinking status (yes/no), education (middle school or below, high school or college or above), residence (urban or rural), marital status, physical activity (continuous), as well as cumulative average vegetable (continuous), fruit (continuous), and energy intake (continuous). Solid lines represent point estimates and ribbons represent 95% CIs. Abbreviations: BMI, body mass index; CI, confidence interval; HR, hazard ratio.

During a median follow-up duration of 6 years (interquartile range, 4 to 13 years) (92,335.5 person-years), 4015 participants developed new-onset hypertension. Multivariable HRs for the risk of new-onset hypertension with increasing tomato consumption were 0.42 (95% CI, 0.37–0.47), 0.51 (95% CI, 0.46–0.57) and 0.82 (95% CI, 0.74–0.92), compared with those in the non-consumers (Table 2). Furthermore, there was a J-shaped relation between dietary tomato intake and new-onset hypertension, with the nadir at approximately 10–13 g/day. (Figure 1, *p* _for non-linearity_ < 0.001).

### 3.3. Sensitivity Analyses

Sensitivity analyses showed no substantial change when we excluded hypertension occurring during the first 2 years of follow-up, and the same results were concluded. Meanwhile, to eliminate the effects of tomato products (canned tomato and ketchup), which are rarely consumed in the Chinese population, we found raw tomato intake was also significantly inversely associated with the risk of new-onset hypertension; the results did not change significantly when the follow-up person-time was calculated from baseline to the first diagnosis of hypertension. When imputed values were used for missing covariates, similar results were found (Table S3 and Supplementary Figure S2). Dividing dietary tomato intake into five groups showed no substantial change (Supplementary Table S4). Finally, the E-values of total dietary tomato consumers ranged from 1.61 (1.36) to 2.35 (1.91) (Supplementary Table S5).

### 3.4. Stratified Analyses by Potential Effect Modifiers

**Figure 2:**
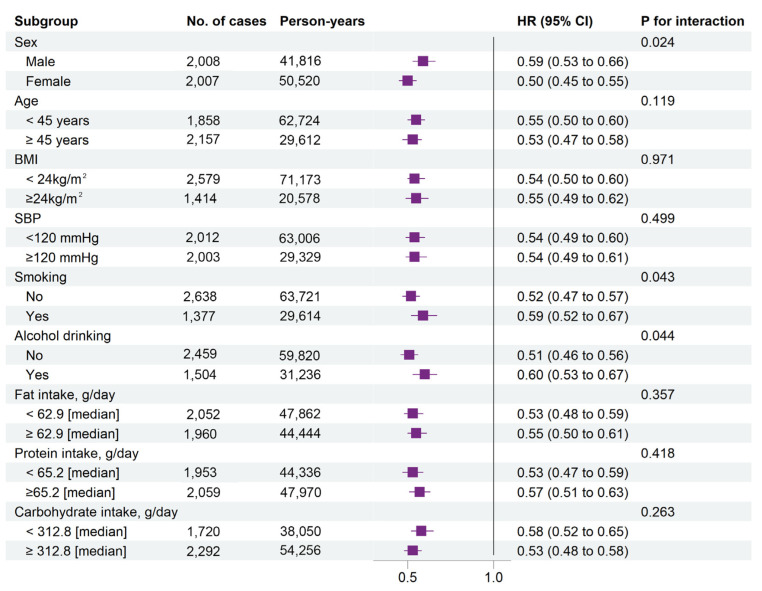
Stratified analyses by potential effect modifiers for the association between dietary tomato intake and new-onset hypertension in various subgroups. Adjusted, if not stratified, for age at baseline (continuous), sex (male/female), BMI (continuous), smoking (yes/no) and drinking status (yes/no), education (middle school or below, high school or college or above), residence (urban or rural), marital status, physical activity (continuous), as well as cumulative average vegetable (continuous), fruit (continuous), and energy intake (continuous). *p* _interaction_ was calculated using the likelihood ratio test. Abbreviations: BMI, body mass index; CI, confidence interval; DBP, diastole blood pressure; HR, hazard ratio; SBP, systolic blood pressure.

**Figure 3:**
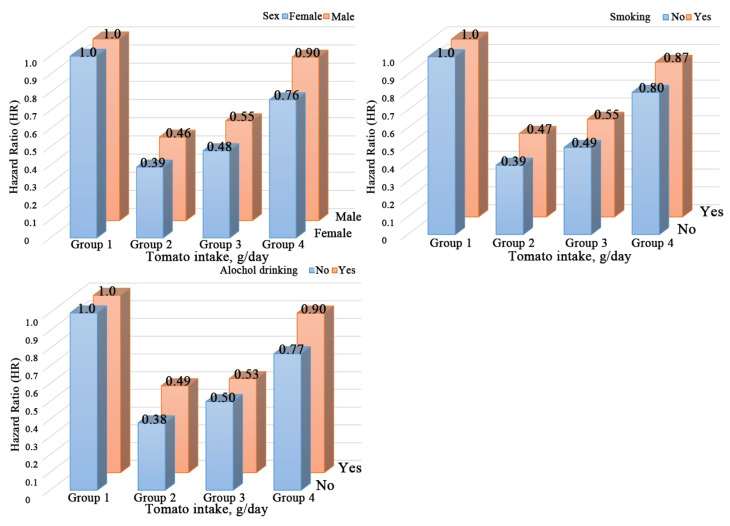
Joint analysis of dietary tomato intake and population characteristics (sex, smoking, and alcohol drinking status) in relation to new-onset hypertension. A: sex; B: smoking; and C: alcohol-drinking status. HRs were adjusted for age at baseline (continuous), sex (male/female), BMI (continuous), smoking (yes/no) and drinking status (yes/no), education (middle school or below, high school or college or above), residence (urban or rural), marital status, physical activity (continuous), as well as vegetable (continuous), fruit (continuous), and energy intake (continuous).

The results of stratified analyses based on potential risk factors were consistent (Figure 2). For total dietary tomato consumption, a weaker inverse association with new-onset hypertension was found in males or those with high-risk lifestyles (alcohol or tobacco use) (*p* _interaction_ < 0.05). In participants who were male and who smoked or drank, the adjusted HR (95% CI) was 0.59 (0.53, 0.66), 0.59 (0.52, 0.67) and 0.60 (0.53, 0.67), respectively, compared with those tomato non-consumers. For participants who were female and who were free of tobacco or alcohol, the adjusted HR (95% CI) was 0.50 (0.45, 0.55), 0.52 (0.47, 0.57) and 0.51 (0.46, 0.56), respectively, compared with those tomato non-consumers (Figure 2 and Figure 3). People who were male or alcohol or tobacco users who consumed more than 33.3 g/day of tomato are not associated with a lower risk of new-onset hypertension, though a J-shape relationship consists of the subgroup mentioned above (Supplementary Table S6 and Supplementary Figure S3). The association between dietary tomato and hypertension was not modified by any other factors, including age, BMI, baseline SBP, fat consumption, protein consumption, or carbohydrate consumption (*p* _interaction_ > 0.05).

## 4. Discussion

To our knowledge, the present study is the first relatively large-scale prospective cohort study to generate and examine evidence on the longitudinal association between tomato consumption and new-onset hypertension events in the general population. Moderate tomato intake versus no consumption was associated with a 49–58% lower risk of new-onset hypertension, independent of other dietary and non-dietary risk factors. Consistently, a novel J-shaped association was found between tomato intake and new-onset hypertension; the rate reduction for new-onset hypertension reached a nadir at approximately 10 to 13 g/day. This tomato-hypertension relation was particularly pronounced in females or alcohol or tobacco non-consumers.

In agreement with previous research, our findings indicate that moderate tomato consumption was inversely associated with total and cause-specific mortality. The prospective study, which included 101,832 participants, showed that participants in the fourth quintile (17.67–32.44 g/day) of tomato intake had a 9% (HR 0.91, 95% CI 0.87–0.95) and 10% (HR 0.90, 95% CI 0.83–0.97) lower risk of total and cardiovascular disease mortality, respectively, than that of participants in the first quintile [21]. Conversely, a previous 2017 meta-analysis of three observational studies suggested that higher tomato or tomato product intake was associated with a non-significantly lower risk of stroke, CVDs and coronary heart disease [22]. This discrepancy may be due to either a limited incidence rate or no adjustment for total energy intake. Of note, the results of this study demonstrated that even when the consumption of tomatoes is lower than in Western countries, benefits can still be observed. The median consumption of dietary tomatoes in the Chinese population was less than 5 g/day [14], whereas, in Western populations, such as the American participants of the PLCO screening trial, the median intake was approximately 13 g/day. 

Additionally, the current study also found a novel non-linear dose-response association between total tomato intake and new-onset hypertension in the general Chinese population. Specifically, 10–13 g/day was best for new-onset hypertension prevention, with the 4 knots RCS model fitting better on the current data. This beneficial effect may be attributed to the healthy constituents of tomatoes, such as lycopene, specific flavonoid compounds, and ascorbic acid. However, excess intake of tomatoes could attenuate the protective effect, which might be related to the excess intake of solanine. In line with a previous prospective study conducted in a western country, a J-shaped association was observed between the consumption of raw tomatoes and total mortality. In this study, 20–40 g/day was observed best for total mortality prevention with the 3 knots RCS model (*p* for non-linearity <0.001) [16]. The discrepancy in optimal intake may be due to the specified number of knots selected for the RCS models. Moreover, a previous short-term RCT in type 2 diabetic patients has suggested that up to 200 g of raw tomato per day for 8 weeks could exert a favorable effect on blood pressure [11]; however, it could not conclude the recommended amount of tomato intake for the prevention of new-onset hypertension in the general population with only a one-dose intervention arm under ideal and controlled circumstances. Therefore, based on the prospective cohort design, the lengthy follow-up period, and repeated dietary assessments, our study indicated that 10–13 g/day, equal to approximately 1 moderate Roma tomato, 2–3 small tomatoes or 5–7 cherry tomatoes per week, was recommended for new-onset hypertension prevention in the general population. It is necessary to validate our findings using other prospective studies in order to reach a consensus with respect to the optimal amount of tomato in the diet. 

Another interesting result from the sub-population analysis in our study indicates that the inverse association between total tomato intake and the risk of new-onset hypertension was stronger in those with low-risk lifestyles of non-smoking or non-drinking. Participants with smoking or drinking habits may have an endothelial dysfunction [23] and chronic inflammation [24] high-risk profile and have a much greater risk of developing hypertension [25,26]. These high-risk lifestyles, such as smoking and drinking, could partially offset the hypertension-reducing effects of tomato intake. Moreover, a previous study showed that participants’ many lifestyles, such as smoking and alcohol consumption, may influence BP in a sex-specific manner [27]. In our study, male participants consumed alcohol more than their female counterparts (the proportion of alcohol consumers was 3260 (62.7) and 672 (11.0) for males and females, respectively), whereas their proportion of smokers was either lower (the proportion of former or current smokers was 3289 (62.8) and 213 (3.4) for males and females, respectively). This disproportional higher ratio of smokers and drinkers in male participants might also contribute to the weaker association between tomato intake and the risk of new-onset hypertension.

The exact biological mechanisms responsible for the protective effect of tomato intake against the development of new-onset hypertension remain largely unknown, although several mechanisms have been proposed. Firstly, this beneficial effect may be partly due to lycopene content (approximately 5.2–23.6 mg/100 g lycopene is present in field-grown tomatoes) [28]. Based on data from the US National Health and Nutrition Examination Survey (NHANES), a previous cross-sectional study shows an inverse relationship between plasma lycopene level and the prevalence of hypertension among overweight and obese individuals [29]. Lycopene is capable of quenching oxygen with high efficiency due to its high number of double bonds [30]. As we know, the role of oxidative stress in hypertension has been confirmed as one of the main pathological mechanisms [31]. Oxidative stress results in an excess of reactive oxygen species that adversely affect vascular function, resulting in decreased nitric oxide synthesis, decreased antioxidant bioavailability, and ultimately vascular dysfunction. In addition to lycopene, tomatoes are also rich in hydrophilic compounds, mainly polyphenols, including flavonoids (chlorogenic acid and rutin) and phenolic acids, which modulate hypertension risk by a number of mechanisms [32,33,34]. Evidence from RCTs has suggested the beneficial effects of hydrophilic compounds on reducing blood pressure in pre-hypertensive males via angiotensin-converting enzyme inhibition. Evidence from a recent network meta-analysis revealed standardized tomato extract significantly decreased SBP compared to a placebo in a population of both healthy volunteers and hypertensive patients [35]. Moreover, potassium is known to lower blood pressure. A 100-g portion of tomatoes contains 212 mg of potassium, which helps in fighting the ill effects of sodium [36]. Recently, a multicenter RCT [37] suggested that heart-healthy Chinese diets with high potassium were also recommended for reducing blood pressure in patients with high blood pressure. However, the possible underlying mechanism is not clear and warrants further investigation.

Our analysis has several strengths. The fact that it is a multi-center, prospective nationwide cohort study with a large sample size in the real world is the main advantage of this study. Another strength is that the long follow-up time and a large number of new-onset hypertension patients provided sufficient power to detect a non-linear association and optimal intake of dietary tomatoes in the general population. Additionally, repeated and validated measurements of the diet with the use of 3-day dietary records were used for dietary intake data [38,39,40]. Moreover, a series of sensitivity analyses was conducted to demonstrate the robustness of our results.

There are several limitations to consider. First, as this study was an observational study, we were unable to determine a causal relationship between tomato and new-onset hypertension. Second, although we have fully adjusted for these potential covariates, there is still a possibility of residuals or confounding. In this case, we use the E-value as a measure of the magnitude of an unmeasured confounding factor, and the result is robust to confounding. Third, the cohort participants were comprised of the general Chinese population; thus, the generalization of the present findings to other demographic or ethnic groups may be limited.

## 5. Conclusions

In conclusion, the association between dietary tomato intake and new-onset hypertension risk was non-linear, following a J shape among general adults, with minimal risk observed at 10–13 g/day. The beneficial effect was more pronounced in females, non-smokers and non-drinkers than their counterparts. These findings provide further support for the current recommendations that moderate tomato consumption is part of a healthy diet for the prevention of new-onset hypertension.

## Figures and Tables

**Table 1:** Baseline population characteristics by total dietary tomato intake ^1^.

Characteristics	Tomato Intake (g/day)					*p*
	Total	Group 1	Group 2	Group 3	Group 4	
Participants, *n*	11,460	7075	1382	1490	1513	
Age, years	41.7 (13.9)	42.2 (14.3)	39.9 (12.4)	40.4 (13.2)	42.1 (13.7)	<0.001
Man, (*n* %)	5234 (45.7)	3281 (46.4)	587 (42.5)	659 (44.2)	707 (46.7)	0.029
BMI, kg/m^2^	22.5 (3.2)	22.4 (3.2)	22.1 (2.8)	22.6 (3.2)	23.0 (3.5)	<0.001
SBP, mmHg	114.1 (11.5)	114.3 (11.6)	111.8 (11.0)	113.1 (11.3)	116.1 (11.0)	<0.001
DBP, mmHg	74.2 (7.8)	74.2 (7.9)	73.1 (8.0)	73.9 (7.9)	75.7 (7.2)	<0.001
Married, (*n* %)	9571 (88.8)	5837 (88.1)	1203 (91.5)	1258 (89.4)	1273 (89.0)	0.004
Education level, (*n* %)						<0.001
Middle school or below	7933 (70.6)	5269 (76.1)	945 (70.1)	899 (61.4)	820 (54.8)	
High school	2458 (21.9)	1277 (18.4)	337 (25.0)	414 (28.3)	430 (28.7)	
College or above	842 (7.5)	379 (5.5)	67 (5.0%)	150 (10.3)	246 (16.4)	
Former or current smoker (*n* %)	3502 (100.0)	2207 (100.0)	431 (100.0)	432 (100.0)	432 (100.0)	0.103
Alcohol consumer (*n* %)	3932 (34.7)	2371 (34.0)	459 (33.6)	533 (36.0)	569 (37.7)	0.023
Urban residence (*n* %)	4410 (38.5)	2184 (30.9)	575 (41.6)	726 (48.7)	925 (61.1)	<0.001
Physical activity METs-h/week, median (IQR)	88.9 (18.0;206.5)	94.0 (16.9;216.0)	99.4 (28.2;223.4)	81.7 (19.9;186.2)	78.3 (16.6;164.4)	<0.001
Energy, kcal/day	2143.8 (564.3)	2165.6 (593.9)	2124.3 (456.5)	2091.5 (496.2)	2111.2 (569.3)	<0.001
Total carbohydrate, % of energy	56.8 (11.3)	58.0 (11.6)	56.4 (9.1)	54.8 (10.0)	53.5 (11.8)	<0.001
Total fat, % of energy	29.8 (10.5)	28.7 (10.8)	30.2 (8.5)	31.7 (9.4)	32.3 (10.9)	<0.001
Total protein, % of energy	12.7 (2.7)	12.5 (2.7)	12.7 (2.3)	13.0 (2.6)	13.6 (3.2)	<0.001
Vegetable intake, g/day	301.1 (136.3)	296.6 (143.4)	293.9 (109.3)	285.0 (106.7)	344.5 (142.1)	<0.001
Fruit intake, g/day	43.1 (87.6)	32.4 (76.4)	40.5 (66.2)	57.7 (87.8)	81.3 (130.6)	<0.001

^1^ Continuous variables are presented as mean (SD) or median (IQR); categorical variables are presented as *n* (%). Information on non-dietary factors was collected at baseline, and dietary data were estimated as cumulative average intake from baseline and follow-up periods. Abbreviations: BMI, body mass index; DBP, diastolic blood pressure; IQR, interquartile range; SBP, systolic blood pressure.

**Table 2:** The relationship of tomato intake with risk of new-onset hypertension.

Tomato Intake, g/day	No. of Cases/Person-Years	Crude Model			Adjusted Model ^1^		
		HR (95% CI)	*p*	*p*_trend_	HR (95% CI)	*p*	*p*_trend_
Four categories							
Group 1	2797/51,308	Ref			Ref		
Group 2	381/16,980	0.39 (0.35, 0.44)	<0.001	<0.001	0.42 (0.37, 0.47)	<0.001	<0.001
Group 3	407/14,384	0.51 (0.46, 0.56)	<0.001		0.51 (0.46, 0.57)	<0.001	
Group 4	430/9663	0.82 (0.74, 0.91)	<0.001		0.82 (0.74, 0.92)	<0.001	
Two categories							
Non-consumers	2700/51,212	Ref			Ref		
Consumers	1152/40,961	0.52 (0.48, 0.55)	<0.001		0.53 (0.49, 0.57)	<0.001	

^1^ Adjusted for age at baseline (continuous), sex (male/female), BMI (continuous), smoking (yes/no) and drinking status (yes/no), education (middle school or below, high school or college or above), residence (urban or rural), marital status, physical activity (continuous), as well as cumulative average vegetable (continuous), fruit (continuous), and total energy intake (continuous). Abbreviations: BMI, body mass index; HR, hazard ratio; CI, confidence interval.

## Data Availability

All data used during the study are available online (https://www.cpc.unc.edu/projects/china, accessed on 5 February 2022).

## Supplementary Materials

The following supporting information can be downloaded at: https://www.mdpi.com/article/10.3390/nu14224813/s1, Figure S1: Flow chart of study participants; Figure S2: Dose-response relationship between the intake of dietary tomato and new-onset hypertension in various sensitivity analyses; Figure S3: Dose-response relationship between intake of total tomato and new-onset hypertension stratified by sex (A), smoking (B) and drinking (C) status; Table S1: The numbers (percentages) of participants with missing covariates; Table S2: Characteristics of participants according to sex; Table S3: Association between dietary tomato intake and new-onset hypertension in various sensitivity analyses; Table S4: The relationship of dietary tomato intake of five categories with risk of new-onset hypertension; Table S5: The E-values of tomato intake with risk of new-onset hypertension, E-value (CI); Table S6: Stratified analyses of the associations between tomato intake and new-onset hypertension.
