# Supplementary material for: J-Shaped Association of Tomato Intake with New-Onset Hypertension in General Adults: A Nationwide Prospective Cohort Study
Source: Nutrients. 2022 Nov 14;14(22):4813. doi: 10.3390/nu14224813 (PMC9696212; doi:10.3390/nu14224813)
Supplement: Supplementary file 1 [file nutrients-14-04813-s001.zip › nutrients-2014112-supplementary.pdf]

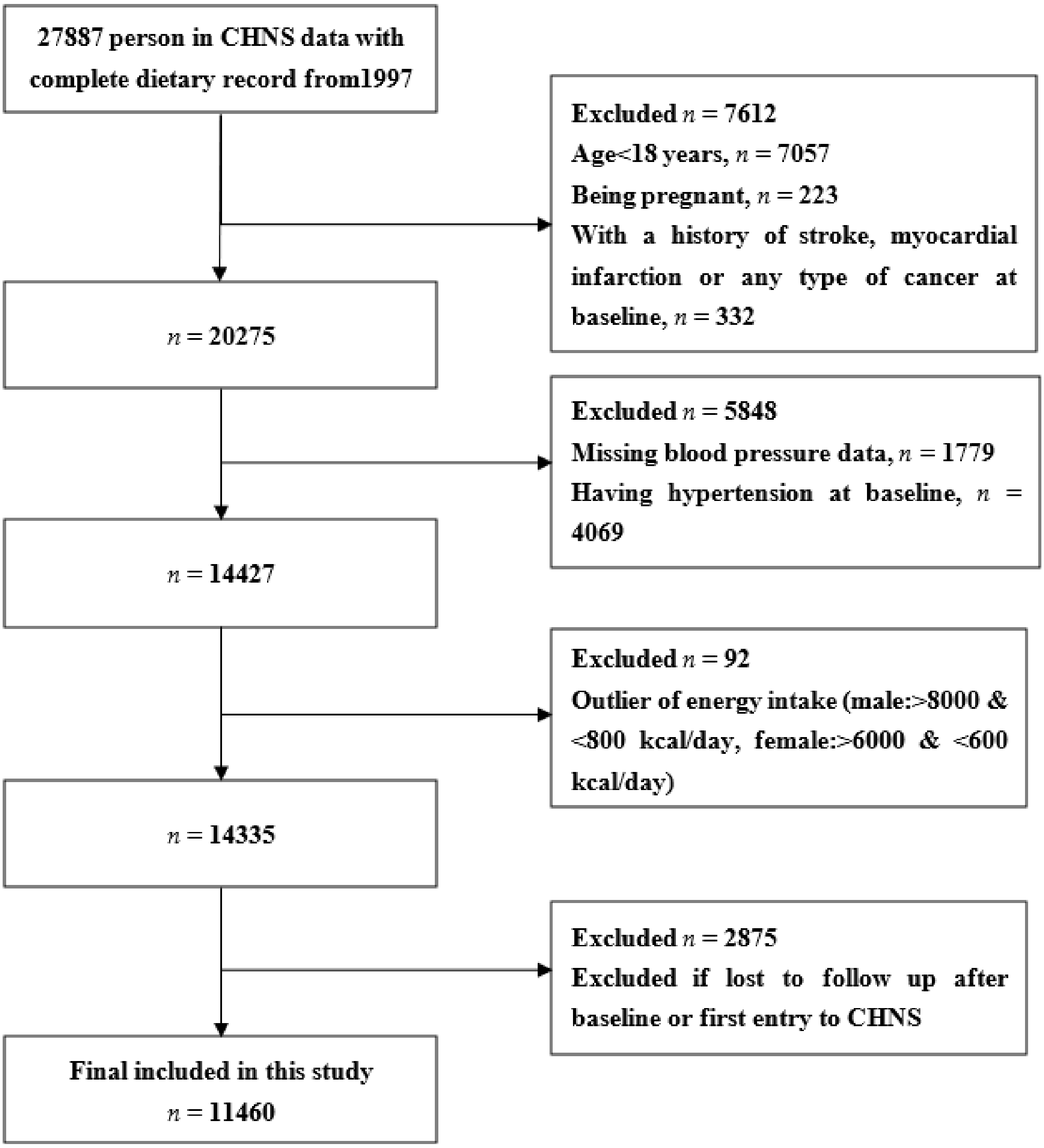

Supplementary Figure S1. Flow chart of study participants.

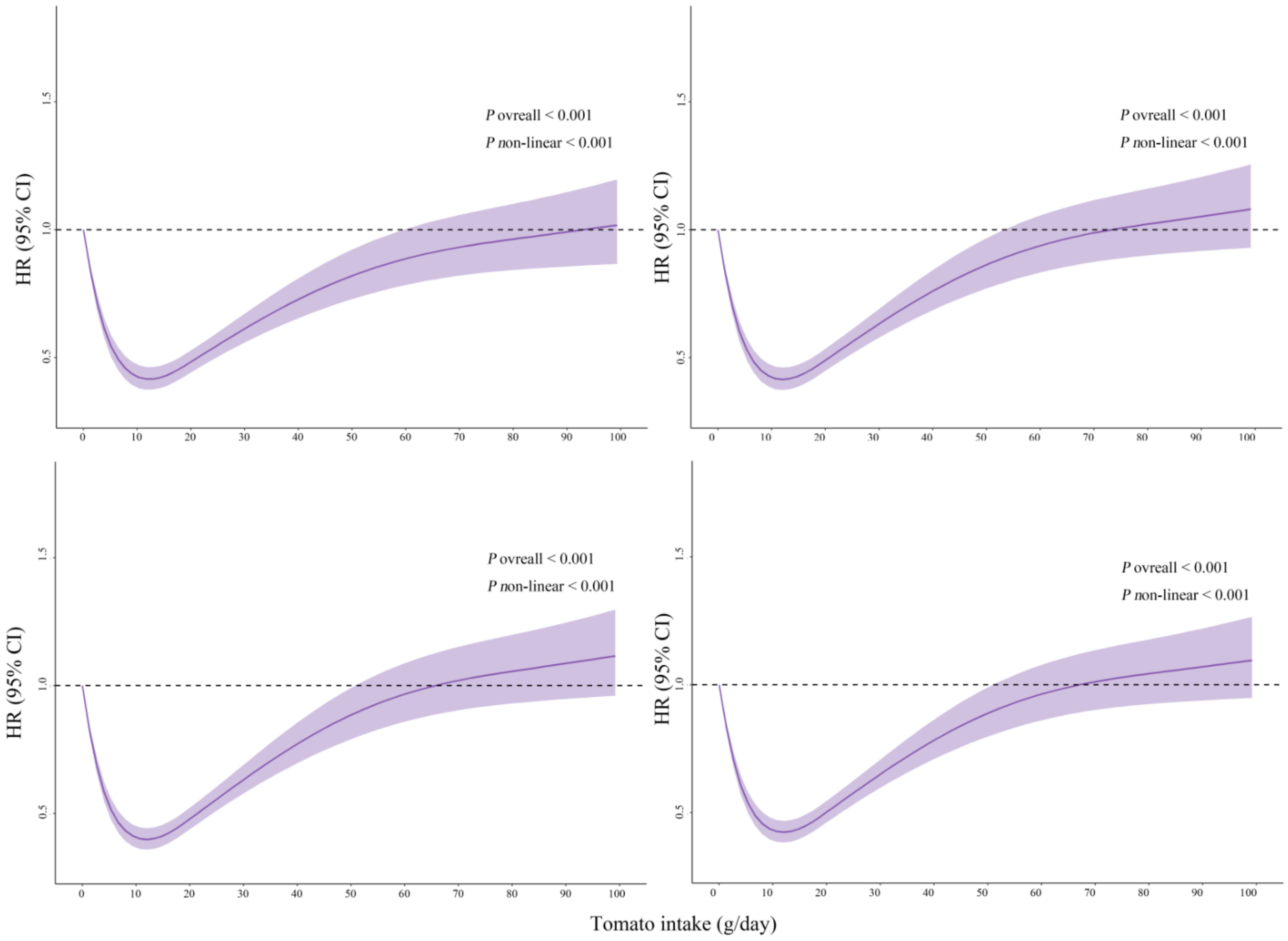

Supplementary Figure S2. Dose-response relationship between the intake of dietary tomato and new-onset hypertension in various sensitivity analyses. A. After excluding new-onset hypertension occurring during the first 2 years of follow-up; B. Dose-response relationship between raw tomato intake with risk of new-onset hypertension; C. The follow-up person-time was calculated as from baseline until the first hypertension diagnosis; D. Use a multiple imputation procedure with 5 rounds of imputation.

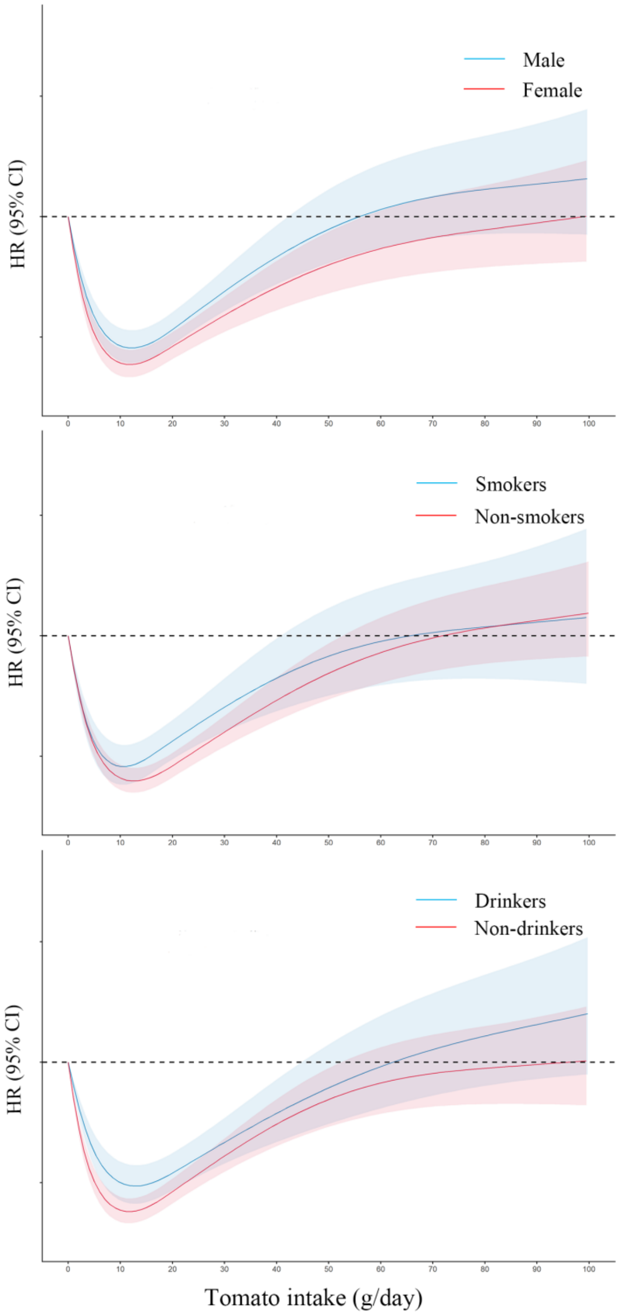

Supplementary Figure S3. Dose-response relationship between intake of total tomato and new-onset hypertension stratified by sex (A), smoking (B) and drinking (C) status. Restricted cubic spline Cox proportional hazard regression model with 4 knots were adjusted for age at baseline (continuous), sex (male/female), BMI (continuous), smoking (yes/no) and drinking status (yes/no), education (middle school or below, high school or college or above), residence (urban or rural), marital status, physical activity (continuous), as well as cumulative average vegetables (continuous), fruits (continuous), and energy intake (continuous), not including the stratification variable for each subgroup. Abbreviations: BMI, body mass index; CI, Confidence Interval; HR, Hazard Ratio.

Supplementary Table S1. The numbers (percentages) of participants with missing covariates.

**Covariates**	***n* (%)**
Physical activity	333 (2.91)
Education level	227 (1.98)
Alcohol consumer	131 (1.14)

Married status	98 (0.85)
BMI	64 (0.56)
Former or current smoker	51 (0.45)
Energy	4 (0.05)
Total carbohydrate intake	4 (0.05)
Total fat intake	4 (0.05)
Total protein intake	4 (0.05)

Supplementary Table S2. Characteristics of participants according to sex *.

Characteristics	Total (*n* = 11460)	Male (*n* = 5234)	Female (*n* = 6226)	*P*
Age, years	41.7 (13.9)	41.8 (14.1)	41.6 (13.8)	0.654
BMI, kg/m^2	22.5 (3.2)	22.4 (3.1)	22.5 (3.3)	0.042
SBP, mmHg	114.1 (11.5)	116.1 (10.6)	112.3 (11.9)	<0.001
DBP, mmHg	74.2 (7.8)	75.5 (7.4)	73.2 (8.0)	<0.001
Married, (*n* %)	9571 (88.8)	4221 (84.1)	5350 (92.9)	<0.001
Education level, (*n* %)				<0.001
Middle school or below	7933 (70.6)	3456 (67.1)	4477 (73.6)	
High school	2458 (21.9)	1262 (24.5)	1196 (19.7)	
College or above	842 (7.5)	433 (8.4)	409 (6.7)	
Former or current smoker, (*n* %)	3502 (30.6)	3289 (62.8)	213 (3.4)	<0.001
Alcohol consumer, (*n* %)	3932 (34.7)	3260 (62.7)	672 (11.0)	<0.001
Urban residence, (*n* %)	7050 (61.5)	3247 (62.0)	3803 (61.1)	0.305
Physical activity, METs-h/week, median [IQR]	88.9 [18.0;206.5]	104.5 [26.9;234.8]	79.3 [15.5;185.7]	<0.001
Energy, kcal/day	2143.8 (564.3)	2343.0 (573.5)	1976.4 (498.4)	<0.001
Total fat, % of energy	29.8 (10.5)	29.1 (10.4)	30.3 (10.5)	<0.001
Total carbohydrate, % of energy	56.8 (11.3)	56.9 (11.5)	56.7 (11.1)	0.465
Total protein, % of energy	12.7 (2.7)	12.6 (2.7)	12.8 (2.8)	<0.001
Vegetable intake, g/day	301.1 (136.3)	313.8 (141.9)	290.3 (130.4)	<0.001
Fruit intake, g/day	0.0 [0.0;50.0]	0.0 [0.0;33.3]	0.0 [0.0;66.7]	<0.001
Tomato intake, g/day, median [IQR]	0.0 [0.0;13.9]	0.0 [0.0;13.3]	0.0 [0.0;13.9]	0.228

* Continuous variables are presented as mean (SD), median (IQR) or categorical variables are presented as *n* (%). Abbreviations: BMI, Body Mass Index; SBP, Systolic Blood Pressure; DBP, Diastole Blood Pressure; CI, Confidence Interval.

Supplementary Table S3. Association between dietary tomato intake and new-onset hypertension in various sensitivity analyses.

Various sensitivity analyses	Dietary tomato intake (g/day) [a]				p trend
	Group 1	Group 2	Group 3	Group 4	
Exclude of new-onset hypertension occurring during the first 2 years of follow-up	1.0	0.42 (0.37,0.47)	0.51 (0.45,0.57)	0.77 (0.69,0.87)	< 0.001
The relationship between raw tomato intake with risk of new-onset hypertension	1.0	0.41 (0.37,0.46)	0.51 (0.45,0.57)	0.82 (0.73,0.92)	< 0.001
The follow-up person-time was calculated as from baseline until the first hypertension diagnosis	1.0	0.40 (0.36,0.45)	0.50 (0.44,0.55)	0.84 (0.75,0.94)	< 0.001
Use a multiple imputation procedure with 5 rounds of imputation	1.0	0.42 (0.37,0.46)	0.53 (0.48,0.59)	0.84 (0.76,0.93)	< 0.001

[a] Multivariate hazard ratios (95%CIs) adjusted for age at baseline (continuous), sex (male/female), BMI (continuous), smoking (yes/no) and drinking status (yes/no), education (middle school or below, high school or college or above), residence (urban or rural), marital status, physical activity (continuous), as well as vegetables (continuous), fruits (continuous), and energy intake (continuous).

Supplementary Table S4. The relationship of dietary tomato intake of five categories with risk of new-onset hypertension.

Total tomato, g/day	No. of cases/ person-years	Crude model			Adjusted model [a]		
		HR (95% CI)	*p*	p_{trend}	HR (95% CI)	*p*	p_{trend}
Five categories							
Group 1	2797/51308	Ref			Ref		
Group 2	296/13485	0.37 (0.33,0.42)	<0.001		0.40 (0.35,0.46)	<0.001	
Group 3	325/11455	0.51 (0.45,0.57)	<0.001		0.50 (0.44,0.57)	<0.001	
Group 4	318/8856	0.59 (0.52,0.67)	<0.001	<0.001	0.6 (0.53,0.68)	<0.001	<0.001
Group 5	318/7231	0.81 (0.72,0.91)	<0.001		0.81 (0.71,0.92)	0.001	

[a]Adjusted for age at baseline (continuous), sex (male/female), BMI (continuous), smoking (yes/no) and drinking status (yes/no), education (middle school or below, high school or college or above), residence (urban or rural), marital status, physical activity (continuous), as well as vegetables (continuous), fruits (continuous), and energy intake (continuous). Abbreviations: HR, Hazard Ratio; CI, Confidence Interval

Supplementary Table S5. The E-values of tomato intake with risk of new-onset hypertension, E-value (CI).

Exposure	Consumers (g/day)			
	Group 1	Group 2	Group 3	Group 4
Total tomato	1 (ref)	2.35 (1.91)	3.031 (2.85)	1.61(1.36)

Abbreviations: CI, Confidence Interval.

Supplementary Table S6. Stratified analyses of the associations between tomato intake and new-onset hypertension.

Sungroup	Group	No. of cases/person-years	Tomato intake, g/day [a]			p for interaction
			HR (95% CI)	*p* value	p_{trend}	
Male	Group 1	1387/23770	ref		<0.001	0.024
	Group 2	182/7202	0.46 (0.39,0.54)	<0.001		
	Group 3	210/6364	0.55 (0.47,0.64)	<0.001		
	Group 4	229/4480	0.90 (0.77,1.05)	0.163		
Female	Group 1	1410/27539	ref		<0.001	
	Group 2	199/9778	0.39 (0.33,0.45)	<0.001		
	Group 3	197/8020	0.48 (0.41,0.57)	<0.001		
	Group 4	201/5182	0.76 (0.65,0.89)	<0.001		
Age < 45 years	Group 1	1254/ 34306	ref		<0.001	0.119
	Group 2	208/11854	0.47 (0.4,0.54)	< 0.001		
	Group 3	208/10264	0.51 (0.44,0.6)	< 0.001		
	Group 4	188/6300	0.84 (0.7,0.99)	0.040		
Age ≥ 45 years	Group 1	1543/17002	ref		<0.001	
	Group 2	173/5126	0.36 (0.3,0.42)	<0.001		
	Group 3	199/4121	0.51 (0.44,0.6)	<0.001		
	Group 4	242/3363	0.83 (0.71,0.96)	0.010		
BMI < 24kg/m²	Group 1	1832/40008	ref		<0.001	0.970
	Group 2	250/13626	0.40 (0.35,0.46)	<0.001		
	Group 3	254/10650	0.55 (0.48,0.63)	<0.001		

	Group 4	243/6890	0.87 (0.75,1.00)	0.057		
BMI ≥ 24kg/m^2	Group 1	949/10994	ref		<0.001	
	Group 2	131/3241	0.45 (0.37,0.55)	<0.001		
	Group 3	149/3632	0.47 (0.39,0.56)	<0.001		
	Group 4	185/2720	0.80 (0.67,0.95)	0.013		
SBP <120 mmHg	Group 1	1382/34889	ref		<0.001	0.500
	Group 2	225/12312	0.43 (0.37,0.50)	<0.001		
	Group 3	231/9865	0.56 (0.48,0.65)	<0.001		
	Group 4	174/5940	0.77 (0.65,0.91)	0.002		
SBP ≥ 120 mmHg	Group 1	1415/16420	ref		<0.001	
	Group 2	156/4667	0.41 (0.35,0.49)	<0.001		
	Group 3	176/4520	0.46 (0.39,0.54)	<0.001		
	Group 4	256/3723	0.83 (0.72,0.97)	0.016		
Smoking: no	Group 1	1850/35122	ref		<0.001	0.043
	Group 2	241/11738	0.39 (0.34,0.45)	<0.001		
	Group 3	266/10074	0.49 (0.43,0.57)	<0.001		
	Group 4	281/6788	0.80 (0.7,0.92)	0.001		
Smoking: yes	Group 1	920/15877	ref		<0.001	
	Group 2	138/5194	0.47 (0.39,0.57)	<0.001		
	Group 3	138/4244	0.55 (0.45,0.66)	<0.001		
	Group 4	140/2748	0.87 (0.72,1.06)	0.174		
Alcohol drinking: no	Group 1	1739/33241	ref		<0.001	0.044
	Group 2	228/11355	0.38 (0.33,0.44)	<0.001		
	Group 3	244/9114	0.50 (0.43,0.58)	<0.001		
	Group 4	248/6109	0.77 (0.67,0.89)	<0.001		
Alcohol drinking: yes	Group 1	1017/17176	ref		<0.001	
	Group 2	149/5392	0.49 (0.41,0.60)	<0.001		
	Group 3	158/5146	0.53 (0.45,0.64)	<0.001		
	Group 4	180/3522	0.90 (0.76,1.07)	0.168		
Fat intake, g/day < 62.9 [median]	Group 1	1501/28170	ref		<0.001	0.357
	Group 2	187/8700	0.40 (0.34,0.47)	<0.001		
	Group 3	194/6711	0.55 (0.47,0.65)	<0.001		
	Group 4	170/4280	0.81 (0.68,0.96)	0.017		
≥ 62.9 [median]	Group 1	1293/23120	ref		<0.001	
	Group 2	194/8279	0.43 (0.37,0.51)	<0.001		
	Group 3	213/7662	0.48 (0.41,0.56)	<0.001		
	Group 4	260/5382	0.82 (0.71,0.95)	0.009		
Protein intake, g/day < 65.2 [median]	Group 1	1416/25518	ref		<0.001	0.418
	Group 2	178/8114	0.41 (0.35,0.48)	<0.001		
	Group 3	176/6396	0.49 (0.42,0.59)	<0.001		
	Group 4	183/4308	0.82 (0.69,0.97)	0.017		
≥65.2 [median]	Group 1	1378/25772	ref		<0.001	
	Group 2	203/8865	0.43 (0.37,0.51)	<0.001		
	Group 3	231/7977	0.54 (0.46,0.63)	<0.001		
	Group 4	247/5356	0.84 (0.72,0.97)	0.020		
Carbohydrate intake, g/day < 312.8 [median]	Group 1	1146/19810	ref		<0.001	0.263
	Group 2	169/6996	0.45 (0.38,0.53)	<0.001		
	Group 3	179/6494	0.50 (0.42,0.59)	<0.001		
	Group 4	226/4750	0.91 (0.78,1.07)	0.256		
≥ 312.8 [median]	Group 1	1648/31480	ref		<0.001	
	Group 2	212/9984	0.41 (0.35,0.47)	<0.001		

	Group 3	228/7880	0.54 (0.47,0.63)	<0.001
	Group 4	204/4912	0.75 (0.64,0.88)	<0.001

[a] Adjusted for age at baseline (continuous), sex (male/female), BMI (continuous), smoking (yes/no) and drinking status (yes/no), education (middle school or below, high school or college or above), residence (urban or rural), marital status, physical activity (continuous), as well as vegetables (continuous), fruits (continuous), and energy intake (continuous). Abbreviations: BMI, Body Mass Index; SBP, Systolic Blood Pressure; HR, Hazard Ratio; CI, Confidence Interval.
